# Independent clinic-based evaluation of point-of-care testing for the screening of *Chlamydia trachomatis*, *Neisseria gonorrhoea* and *Trichomonas vaginalis* in women-at-risk in Australia, Guatemala, Morocco, and South Africa

**DOI:** 10.1186/s12879-024-09018-4

**Published:** 2024-03-04

**Authors:** Mark Shephard, Susan Matthews, Ranmini Kularatne, Kelly Andrewartha, Karel Blondeel, Christian Alvarez, Elsy Camey, Amina Hançali, Etienne Müller, Aurelie Haw, Hicham Oumzil, Daniel Golparian, Dorian E Ramirez, James Kiarie, Firdavs Kurbonov, Massimo Mirandola, Rosanna W Peeling, Ronaldo Silva, Soe Soe Thwin, Magnus Unemo, Igor Toskin

**Affiliations:** 1 International Centre for Point-of-Care Testing, Flinders University, Bedford Park, South Australia Australia; 2 Centre for HIV & STI, National Institute for Communicable Diseases, Johannesburg, South Africa; 3 Department of Sexual and Reproductive Health and Research, World Health Organization, Geneva, Switzerland; 4 Faculty of Medicine and Health Sciences, Ghent University, Ghent, Belgium; 5 Sida y Sociedad, ONG (SISO), Escuintla, Guatemala; 6 National Institute for Health, Rabat, Morocco; 7 Médecins sans Frontières, Khayelitsha, Cape Town, South Africa; 8 WHO Collaborating Centre for Gonorrhoea and other STIs, Department of Laboratory Medicine, Faculty of Medicine and Health, Örebro University, Örebro, Sweden; 9 Facultad de Ciencias Médicas, Universidad de San Carlos de Guatemala, Guatemala City, Guatemala; 10 Infectious Diseases Section, Department of Diagnostics and Public Health, University of Verona, Verona, Italy; 11 School of Sport and Health Sciences, University of Brighton, Brighton, United Kingdom; 12 Department of Clinical Research, London School of Hygiene and Tropical Medicine, London, United Kingdom; 13 Institute for Global Health, University College London, London, United Kingdom

**Keywords:** Point-of-care testing, Sexually transmitted infections, GeneXpert, Multi-country, Sensitivity, Specificity

## Abstract

**Background:**

In 2018, the World Health Organization commenced a multi-country validation study of the Cepheid GeneXpert for a range of molecular-based point-of-care (POC) tests in primary care settings. One study arm focused on the evaluation of POC tests for screening ‘women at risk’ for chlamydia (CT), gonorrhoea (NG) and trichomonas (TV) in four countries – Australia, Guatemala, Morocco and South Africa.

**Methods:**

Study participants completed a pre-test questionnaire which included demographics, clinical information and general questions on POC testing (POCT). Two vaginal swab samples (either self-collected or clinician collected) from each patient were tested on the GeneXpert at the POC and at a reference laboratory using quality-assured nucleic acid amplification tests (NAATs).

**Results:**

One thousand three hundred and eighty-three women were enrolled: 58.6% from South Africa, 29.2% from Morocco, 6.2% from Guatemala, and 6.0% from Australia. 1296 samples for CT/NG and 1380 samples for TV were tested by the GeneXpert and the reference NAAT. The rate of unsuccessful tests on the GeneXpert was 1.9% for CT, 1.5% for NG and 0.96% for TV. The prevalence of CT, NG and TV was 31%, 13% and 23%, respectively. 1.5% of samples were positive for all three infections; 7.8% were positive for CT and NG; 2.4% were positive for NG and TV; and 7.3% were positive for CT and TV. Compared to reference NAATs, pooled estimates of sensitivity for the GeneXpert tests were 83.7% (95% confidence intervals 69.2-92.1) for CT, 90.5% (85.1-94.1) for NG and 64.7% (58.1-70.7) for TV (although estimates varied considerably between countries). Estimates for specificity were ≥96% for all three tests both within- and between-countries. Pooled positive and negative likelihood ratios were: 32.7 ([CI] 21.2-50.5) and 0.17 (0.08-0.33) for CT; 95.3 (36.9-245.7) and 0.10 (0.06-0.15) for NG; and 56.5 (31.6-101.1) and 0.35 (0.27-0.47) for TV.

**Conclusion:**

This multi-country evaluation is the first of its kind world-wide. Positive likelihood ratios, as well as specificity estimates, indicate the GeneXpert POC test results for CT, NG and TV were clinically acceptable for ruling in the presence of disease. However, negative likelihood ratios and variable sensitivity estimates from this study were poorer than expected for ruling out these infections, particularly for TV.

**Trial registration:**

Ethics approval to conduct the ProSPeRo study was granted by the WHO Ethics Review Committee, as well as local ethics committees from all participating countries.

## Background

Globally, the incidence of sexually transmitted infections (STI) continues to present major contemporary health challenges. STIs can be caused by a range (up to 30 species) of bacterial, parasitic or viral pathogens. In 2020, three common bacterial pathogens – *Chlamydia trachomatis* (CT), *Neisseria gonorrhoeae* (NG) and *Treponema pallidum* (syphilis) – together with the parasitic pathogen *Trichomonas vaginalis* (TV) accounted for 374 million new STI cases globally; meaning these STIs were collectively responsible for more than one million new infections every day during 2020 [1–3]. If left untreated, these four infections can lead to debilitating long-term complications; for example, chlamydia, gonorrhoea and trichomoniasis may cause pelvic inflammatory disease in women; gonorrhoea and syphilis may lead to greater risk of acquiring human immunodeficiency virus (HIV); and syphilis (as well as other STIs) may result in an increased risk of stillbirth, neonatal death, low birth weight, prematurity and congenital abnormalities through mother-to-child transmission.

Chlamydia, gonorrhoea, syphilis and trichomoniasis are generally curable with targeted antibiotics. However, these STIs are often asymptomatic or, if symptoms are present, they are often non-specific. Low- and middle-income countries (LMICs) generally rely on syndromic management as the first line of treatment for STIs, but this approach frequently leads to a missed diagnosis if patients are asymptomatic or overtreatment/mistreatment with antibiotics (given the specific infectious agent cannot be identified) [4]. Indeed, antimicrobial resistance (AMR) has now become a major issue globally for the treatment of gonorrhoea, with this bacterial species now resistant to multiple antibiotic agents including fluoroquinolines, macrolides, sulphonamides, penicillin, tetracyclines and even extended spectrum cephalosporins [5, 6].

Laboratory tests are available to detect many of these STIs in high income countries (HIC) and they are especially useful for the diagnosis of asymptomatic patients; however, the cost of performing these tests is often prohibitive. In LMICs, these tests are largely unavailable, while in remote communities geographically isolated from routine laboratory testing, long turnaround times for return of test results and the concomitant loss-to-follow-up of patients makes laboratory testing and linkage to care challenging.

Molecular testing for CT and NG has been available in the laboratory setting for two decades; however, until recently, diagnostic technologies for point-of-care testing (POCT) have focused on lateral flow immunochromatographic strip methods which exhibited poor sensitivity and specificity [7–10]. In 2013, molecular-based options (such as nucleic acid amplification testing [NAAT]), suitable for the detection of CT and NG at the point-of-care, first appeared on the diagnostic market [10].

The key concern regarding the use of molecular POCT methods was whether they demonstrated sound analytical and clinical performance, equivalent to that exhibited by the laboratory based NAATs. Standard practice to answer this question is to conduct a method evaluation where a range of patient samples (both negative and positive of varying infectious loads) are tested in parallel by the POCT (test) method and the laboratory NAAT (reference) method. The comparative performance of the POCT versus the laboratory method can then be assessed using statistical indicators which (for qualitative tests) include *inter alia* sensitivity (Sn), specificity (Sp), likelihood ratio (LR) or diagnostic odds ratio (DOR) [11]. Such evaluations should occur not only in a controlled laboratory environment but, where possible, in the field; that is, in the clinical setting (for example, primary care) where patient testing will routinely occur and where testing is performed by (non-laboratory) health professionals working in a mostly busy setting (rather than by laboratory technicians/scientists) [12].

During the past five years, the WHO commenced a multi-country validation study of the performance of the GeneXpert device for a range of molecular-based POC tests in a variety of primary care settings. The so-named ProSPeRo study (Project on Sexually Transmitted Infection Point-of-Care Testing) was administered by the Sexual and Reproductive Health and Research Department of the WHO. One arm of the study focused on screening ‘women at risk’ for CT, NG and TV in four countries – South Africa, Guatemala, Morocco and Australia (the latter conducting TV testing only) – with parallel samples measured both by on-site POC testing on the GeneXpert in a primary care setting by non-laboratory-trained personnel and by the best available and sufficiently evaluated reference NAAT in a laboratory.

This paper reports on the findings of this evaluation, discusses demographic and operational characteristics, and provides commentary on factors that have contributed to between-country variability in observed analytical performance. These findings can be applied to improve POC testing for STIs using the GeneXpert.

## Methods

### Countries and sites involved

Four countries participated in the field evaluation of the GeneXpert’s performance for measuring CT, NG and TV in the ’women at risk’ arm of PRoSPeRo – South Africa, Guatemala, Morocco and Australia.

Three countries (Guatemala, Morocco and South Africa) performed clinic-based testing for all three pathogens. In Guatemala, testing was conducted at an STI clinic in a regional based hospital in the city of Escuintla; in Morocco at a centre for free and voluntary HIV and STI testing in the capital city of Rabat; and in South Africa at a youth clinic in the Khayelitsha district near Cape Town. Australia performed TV testing only. Three Australian sites were initially engaged but, due to workflow issues at two of the services, the study was completed largely at one remote Indigenous health clinic in an offshore island off the north coast of the Northern Territory. The study took place in these countries either just prior to or during the COVID-19 pandemic.

A second site from Guatemala – an STI/HIV clinic from a major hospital in the city of Guatemala – participated in the study from October 2022 to April 2023; the COVID-19 pandemic having precluded this site starting in the study until after the pandemic had subsided. The Guatemala 2 site underwent similar training in GeneXpert STI testing to the other sites but did not perform QC or EQA testing as supplies of these quality materials were exhausted during the main study period. As such, the data subset from this site will be described separately in this paper.

### Patient cohorts tested

Women aged 18 years or over who were considered ‘at risk’ for STIs were tested as part of this validation study. Women ‘at risk’ were defined as: women reporting unprotected sexual intercourse with more than one partner in the last 12 months; women reporting a past history of STIs; women performing sex work and/or Australian Indigenous women living in remote Aboriginal communities [13]. Prior to having their POC test performed and following their informed consent, each participant was assigned a unique project identification number and was interviewed by a clinic nurse and asked a series of 14 questions. Participant responses were transcribed onto individual, de-identified case report forms (CRF). The pre-test question set included demographic information; general questions about POCT and how long patients would be willing to wait for a POC test result; aspects of their clinical examination; and questions, where appropriate, about history of any STIs.

Two vaginal swab samples were then collected from each patient. In South Africa, these samples were self-collected; in Morocco and Guatemala, they were collected by the provider/clinician responsible for the patient; and in Australia, approximately one-quarter were self-collected and three-quarters were provider-collected. One of the swabs was collected using a Cepheid Xpert specimen collection kit (Cepheid, Sunnydale, California, USA) and used for on-site analysis on the GeneXpert. The second swab was collected using a collection device/transport media specified by the appropriate reference laboratory and shipped for either immediate laboratory testing or storage at a minimum of -20^O^C for up to three months until analysis. There was no order specified for the vaginal swab collection, with regard to the allocation for GeneXpert testing or laboratory referral.

### Test methods for POCT and reference laboratories

The GeneXpert system (Cepheid, Sunnydale, California, USA) was used at the clinics to test for CT, NG and TV. The GeneXpert is fully automated, cartridge-based, and integrates sample processing, cell lysis, purification, nucleic acid amplification, and detection. The real-time Xpert® CT/NG assay (Cepheid) simultaneously detects a single gene target (CT1) for CT and two gene targets (NG2 and NG4) for NG and generates a simultaneous qualitative result (negative or positive) for each gene target and an overall result of ‘not detected’ or ‘detected’ for CT and NG in 90 min. Both NG targets need to be detected for the NG result to be reported as ‘detected’. Separate test cartridges (Xpert® TV assay [Cepheid]) were used for the qualitative detection of TV (one gene target) with a result turnaround time of 60 min. All GeneXpert testing and interpretation of results were performed in accordance with the manufacturer’s instructions (Cepheid).

In terms of the reference laboratory test, samples from Guatemala and Morocco were sent to the WHO Collaborating Centre for Gonorrhoea and Other STIs in Sweden for determination of CT, NG and TV on the Hologic Panther device, while samples from South Africa were analysed at an in-country reference laboratory also using the Hologic Panther. The Aptima Combo 2 (CT, NG; Hologic) and Aptima TV assays (Hologic) were used in both countries. In Australia, TV samples were analysed by a reference laboratory in Perth, Western Australia which used both the Cobas 4800 device and the Hologic Panther during the study. All reference testing and interpretation of results followed the manufacturer’s instructions (Hologic, Roche).

### Training and quality systems

Between countries, there were local differences in the delivery of GeneXpert POCT training and the health professional status of the operators trained (Table 1). Nonetheless, all operators were deemed competent by study organisers to perform GeneXpert testing. Except for Guatemala site 2, each site was required to routinely conduct quality control and quality assurance testing to monitor the analytical performance for the Xpert CT/NG or TV assays [14].

**Table 1:** Summary of how training was delivered in each participating country

Comparative Criteria	Australia	South Africa	Guatemala Site 1	Guatemala Site 2	Morocco
Institution type	Remote primary care health service (funded by Northern Territory Government)	Primary care clinic in Khayelitsha, Cape Town (the second largest township in South Africa)	Regional hospital with STI clinic in the city of Escuintla	HIV and STI clinic, from the Ministry of Health, in the city of Guatemala	Centre de formation et de depistage anonyme et gratuit (CIDAG) (Anonymous and free screening centre) Association de Lutte contre le Sida (ALCS) Rabat (Moroccan non-government organisation fighting against HIV/AIDS)
Number of operators conducting patient testing	1	5	1	1	3
Operator profession	Regional Sexual Health Co-ordinator	Professional nurses	Lab technician	Lab technician	Physicians
Level of experience with GeneXpert prior to training	None	None	None	Experience from a prior study	None
Duration of training:	4 h	Over three days	4 h	4 h (3 theoretical, 1 practical)	2 days
Assessment of training	Written and practical	Written and practical	Written and practical	Written and practical	Written and practical
Who delivered training?	ICPOCT senior scientist	Local Cepheid technical expert	Local Cepheid representative for the GeneXpert (Labymed); delivered in-person	Local Cepheid representative for the GeneXpert (Labymed); delivered on-line	Local Cepheid representative for the GeneXpert POC Scientist (Principal investigator [PI] for study)
Health literacy of operators	No translation of resources required	No translation of resources required	Resources translated from English to Spanish	Resources translated from English to Spanish	Resources translated from English to French
Duration of testing during the project	17 months (June 2018 to Nov 2019)	17 months (Oct 2018 to March 2020)	2 months (Jan 2020 to Feb 2020)	6 months (Oct 2022 to Apr 2023)	24 months (Jan 2020 to Jan 2022)

### Data handling

The patient’s POC test results (including information on date of test and lot number and expiry date of cartridges) were transcribed manually from the GeneXpert onto the individual’s CRF by the POCT operator. Test results were reported as either ‘positive’ (detected), ‘negative’ (not detected), ‘invalid’, ‘error’ or ‘no result’. The latter three result options indicated that the test was unsuccessful and the GeneXpert did not produce a valid result. An ‘invalid’ result indicated that the in-built Sample Processing Control (SPC) and/or Sample Adequacy Control (SAC) failed, the sample was not properly processed, PCR was inhibited, or the sample was inadequate. An ‘error’ result indicated that the internal Probe Check Control (PCC) failed and the assay was aborted because either the reaction tube was filled improperly, a reagent probe integrity problem was detected, pressure limits were exceeded, or a valve positioning error was detected. A ‘no result’ indicated that insufficient data were collected; for example, the operator stopped a test in progress. Samples that were initially reported as ‘invalid’, ‘error’ or ‘no result’ were repeated if sample volume allowed; if a valid result was obtained on repeat, then this result was reported for this patient.

Each site also had a designated data entry operator/clerk who entered the information from the CRF into the WHO clinical trial management system, Open Clinica. In Australia, a data entry clerk and scientist also crosschecked each result reported on the CRF with the POCT result from the GeneXpert laptop (via remote access) before entry into Open Clinica. In South Africa, a data clerk entered only the information documented on the CRFs but did not crosscheck the GeneXpert result. Results however were double-checked post facto by the WHO study organisers and a small number (approximately 20–30) of manual transcription errors were identified and corrected prior to final data analysis. Crosschecking of results did not occur in Morocco or Guatemala.

Results from NAAT performed by the reference laboratories were extracted from the respective laboratory information systems, documented in a Microsoft Excel spreadsheet, merged into Open Clinica, and then crosschecked. They were reported as either ‘positive’ (detected), ‘negative’ (not detected), ‘missing’, ‘invalid’, or ‘error’. A ‘missing’ sample was not included in the paired method comparison because either the sample arrived at the laboratory in a state unsuitable for analysis or was misplaced during transport. ‘Invalid’ and ‘error’ codes on the Hologic occurred when the platform could not provide a result for the sample.

### Sub-analysis of results – effect of bloodstained samples on performance characteristics

Following the initial analysis of performance characteristics observed on the GeneXpert, a separate audit of samples from South Africa was undertaken to determine how many samples were blood-stained and the potential impact of this parameter on the reported sensitivity and specificity.

### Ethics approval

Ethics approval for the core protocols of the CT, NG and TV arms of the ProSPeRo study was obtained by the WHO Ethics Review Committee (ERC). Locally adapted site-specific protocols were approved by the respective local ethics committees and by the ERC.

## Results

### Population profile

A total of 1383 women were enrolled in the study from the four participating countries, 58.6% (810/1383) of participants were from South Africa; 29.2% (404/1383) from Morocco; 6.2% (86/1383) from Guatemala (Site 1); and 6.0% (83/1383) from Australia.

Slightly less than half of the participants (48.9%) were aged between 20 and 24 years, 13% from 18 to 19 years, 8% from 25 to 29 years, 7% from 30 to 34 years, 6% from 35 to 39 years, 8% from 40 to 44 years, 4% from 45 to 49 years and 6% from 50 to 69 years. The median age of the 1383 participants was 23.0 years (interquartile range (IQR) 21–33 years); however, median ages (IQR) for each country varied considerably: South Africa 22 years (IQR 20–23 years), Morocco 40 years (IQR 32–46), Guatemala (Site 1) 27 years (IQR 23–32) and Australia 33 years (IQR 26–42). A small proportion of the participants (0.72%) were pregnant at the time of enrolment.

When asked the question: ‘would you be willing to wait for [your POC test] results at the clinic, directly after the tests are performed?’, almost 70% (69.9%) of the 1383 participants responded in the affirmative. When then asked: ‘how long would you be willing to wait?’, just over 39% of those respondents stated they would be prepared to wait for up to 20 min for their result, 28% up to 30 min, 13% up to an hour, 12% up to two hours, and 7% did not know or specified other waiting times.

In relation to past STI history, 4.2% of the 1383 participants indicated they had been previously diagnosed with gonorrhoea, 1.7% with chlamydia, 20.8% with HIV, 6.8% with syphilis, 4.4% with trichomoniasis, and 50% with other STI symptoms (most notably vaginal discharge [>80%] and papilloma [4%]). Between countries, gonorrhoea was the most prevalent past infection in Morocco (11%); chlamydia in Australia (12%); HIV in Morocco (25%) and South Africa (23%); syphilis in Morocco (19%) and Australia (14%); and trichomoniasis in Australia (49%).

Moreover, 8.9% of 1258 respondents reported having taken any antibiotics in the past three weeks (prior to POCT), with rates ranging from 4.7% in Guatemala (Site 1) to 15% in Morocco.

Regarding signs and symptoms of infection, 7% of 1383 respondents complained of dysuria (painful urination), 24% reported vaginal discharge, 12% experienced vulva itching or burning, and 7% reported ‘other symptoms’, mainly papillomas. Just over 43% of participants also underwent a physical examination.

### Results of method comparison

A total of 1296 samples for CT/NG and 1380 samples for TV were tested for parallel analysis by the GeneXpert (POCT) and by the reference NAAT across the three countries conducting all these tests. For CT, 24 unsuccessful tests were reported on the GeneXpert (11 ‘invalid’, 9 ‘error’ and 4 ‘no result’), while 16 unsuccessful tests were documented by the reference laboratory (11 ‘missed samples’, one ‘error’ and 4 ‘invalid’). For NG, 19 unsuccessful tests were reported by the GeneXpert (11 ‘invalid’, 5 ‘error’ and 3 ‘no result’) and 22 unsuccessful tests by the laboratory (11 ‘missed’ 7 ‘error’ and 4 ‘invalid’). For TV, there were 13 unsuccessful GeneXpert tests (5 ‘invalid’, 7 ‘error’ and 1 ‘no result’) and 22 unsuccessful laboratory tests (14 ‘missed’ and 8 ‘invalid’). Overall, the rate of unsuccessful tests on the GeneXpert was 1.9% for CT, 1.5% for NG and 0.96% for TV, while for the laboratory, the unsuccessful test rate was 1.2% for CT, 1.7% for NG and 1.6% for TV.

In total, there were 1255 valid (paired) tests for CT, 1256 for NG and 1345 for TV available for subsequent concordance analysis. Based on the results from the reference NAATs, the overall prevalence of CT, NG and TV in the ‘women at risk’ populations surveyed was 31%, 13% and 23% respectively (Table 2). In terms of co-infection rates, 1.5% of samples (19/1273) were positive for CT, NG and TV; 7.8% (99/1277) were positive for both CT and NG; 2.4% (31/1274) were positive for both NG and TV; and 7.3% (93/1280) were positive for both CT and TV.

**Table 2:** Estimated prevalence^a^ of STIs in populations surveyed, based on reference laboratory testing

Country	Chlamydia	Gonorrhoea	Trichomoniasis
Morocco	20.3% (81/399)	2.8% (11/399)	39.5% (158/400)
Guatemala (Site 1)	19.8% (17/86)	2.3% (2/86)	15.1% (13/86)
South Africa	38.2% (305/799)	20.0% (159/794)	15.6% (124/795)
Australia	Test not performed	Test not performed	17.5% (14/80)
Pooled Prevalence	31.1% (403/1284)	13.4% (172/1279)	22.7% (309/1361)

*STIs = *Sexually transmitted infections, *Chlamydia* = *Chlamydia trachomatis*, *Gonorrhoea*  = *Neisseria gonorrhoeae*, *Trichomoniasis =* *Trichomonas vaginalis*

^a^Estimate of prevalence based on positive results reported by the reference laboratory/total number of valid tests performed by the laboratory

Compared to the reference NAATS, the sensitivity and specificity (with 95% confidence intervals) observed for the GeneXpert both across and between the participating countries is shown in Table 3. The reported specificity both within-and between-countries was excellent (≥ 97% for CT, ≥ 98% for NG and ≥ 98% for TV), with narrow confidence intervals for all three tests. Overall (pooled) estimates of sensitivity were 83.7% (95% confidence intervals [CI] 69.2–92.1) for CT, 90.5% (85.1–94.1) for NG and 64.7% (58.1–70.7) for TV. However, these estimates of sensitivity also varied considerably between country; for example, for CT, sensitivity ranged from 70.1% (CI 58.6–80.0) in Morocco to 91.4% (87.6–94.3) in South Africa and, for TV, from approximately 59.7% (51.5–67.7) in Morocco to 91.7% (61.5–99.8) in Guatemala (Site 1). For the GeneXpert NG assay, which has two gene targets, the between-country variability of sensitivity estimates was less, ranging from 90.4% (84.6–94.5) in South Africa to 100% in Guatemala (Site 1), but confidence intervals were wide.

**Table 3:** Calculated sensitivity and specificity (and 95% confidence intervals) for the GeneXpert (using random effects model)

**Chlamydia**			
* Country*	*Valid Cases*	*Sensitivity (95% Confidence Intervals)*	*Specificity (95% Confidence Intervals)*
Morocco	383	70.1% (58.6–80.0)	97.7% (95.3–99.1)
Guatemala (Site 1)	86	82.4% (56.6–96.2)	100% (94.8–100)
South Africa	785	91.4% (87.6–94.3)	96.9% (95.0-98.3)
Pooled estimate	1254	83.7% (69.2–92.1)	97.4% (96.1-98.3%)
**Gonorrhoea**			
* Country*	*Valid Cases*	*Sensitivity (95% Confidence Intervals)*	*Specificity (95% Confidence Intervals)*
Morocco	388	90.9% (58.7–99.8)	99.7% (98.5–100)
Guatemala (Site 1)	86	100% (15.8–100)	98.8% (93.5–100)
South Africa	779	90.4% (84.6–94.5)	98.4% (97.1–99.2)
Pooled estimate	1253	90.5% (85.1–94.1)	99.1% (97.6–99.6)
**Trichomoniasis**			
* Country*	*Valid Cases*	*Sensitivity (95% Confidence Intervals)*	*Specificity (95% Confidence Intervals)*
Morocco	393	59.7% (51.5–67.6)	98.8% (96.4–99.8)
Guatemala (Site 1)	84	91.7% (61.5–99.8)	100% (95.1–100)
South Africa	786	66.1% (57.1–74.4)	98.8% (97.6–99.5)
Australia	69	84.6% (54.6–98.1)	100% (94.6–100))
Pooled estimate	1332	64.7% (58.1–70.7)	99.0% (98.1–99.4)

Positive and negative likelihood ratios (LR + and LR- respectively) were also calculated, given these diagnostic tests, unlike predictive values, are independent of prevalence [11]. LRs of greater than 10 or less than 0.1 are generally considered to provide strong evidence to either rule in (LR+) or rule out (LR-) disease diagnoses, respectively [15]. For CT, the LR + and LR- were 32.7 (95% CI 21.2–50.5) and 0.17 (0.08–0.33) respectively; for NG, they were 95.3 (36.9-245.7) and 0.10 (0.06–0.15); and for TV 56.5 (31.6-101.1) and 0.35 (0.27–0.47).

### Subset of data from Guatemala Site 2

The subset of data from Guatemala Site 2 provided an interesting contrast with the main study data as POC tests conducted at this site were performed without the use of quality surveillance using QC and EQA testing. There were 678 valid (paired) tests for CT and NG and 622 for TV available for subsequent concordance analysis from Guatemala Site 2. Table 4 shows the prevalence, sensitivity and specificity for this subset. Estimates for all parameters were similar to Guatemala Site 1, except for TV sensitivity which was much poorer for Site 2 (60%) compared to 91.7% (Site 1). Overall, if the Guatemala Site 2 data was included in the pooled estimates of sensitivity, specificity and likelihood ratio, there was little change to these parameters.

**Table 4:** Prevalence and diagnostic performance observed at Guatemala Site 2

Parameter	Chlamydia	Gonorrhoea	Trichomoniasis
Prevalence	7.6%	1.0%	8.0%
Sensitivity (CI)	84.6% (71.9–93.1)	100% (59.0-100)	60.0% (45.2–73.6)
Specificity (CI)	99.0% (97.9–99.7)	100% (99.5–100)	100% (99.4–100)
Change to pooled estimates:			
Sensitivity (CI)	83.6% (72.8–90.6)	90.9% (85.7–94.4)	64.0% (58.9–68.9)
Specificity (CI)	98.3% (96.9–99.1)	99.6% (98.1–99.9)	99.6% (97.5–100)
Likelihood Ratio + (CI)	48.7 (27.8–85.5)	240.3 (47.6-1214.8)	176.6 (25.5–1224.0)
Likelihood Ratio - (CI)	0.17 (0.10–0.29)	0.09 (0.06–0.15)	0.36 (0.31–0.42)

## Discussion

Prior to the present WHO-led ProSPeRo study, there have been a limited number of published studies on the comparative performance of the GeneXpert system for STI testing, especially for TV: with one field-based study (TTANGO; Test, Treat ANd GO) and several laboratory-based studies having been conducted in Australia and the USA [16–22].

In the Australian studies, a laboratory evaluation involving 372 characterised CT or NG bacterial strains concluded the GeneXpert CT/NG POCT cartridge was highly sensitive and specific for these infectious agents [16]. A field evaluation of the CT/NG test was then conducted In Australia as part of a randomised controlled trial called TTANGO undertaken in 12 remote Aboriginal communities and involving 2486 self-collected urine or lower vaginal swabs [17, 19]. The overall concordance between the GeneXpert POC test and laboratory based NAATs was 99.4% for CT and 99.9% for NG [17].

In the US, a multicentre evaluation involving 1,722 females and 1,387 males was conducted in a range of clinic settings (either obstetrics and gynaecological, STI, public health, teen, or family planning clinics) [18]. Vaginal swabs and urine samples from females and urine samples from men were tested in the laboratory, both on the GeneXpert and one of two NAAT reference tests. The sensitivity for CT testing on endocervical and vaginal samples from women as well as urine samples from men and women was 97.4% or better, while specificities for corresponding sample types were all greater than 99.4%. For NG, sensitivities on the same specimens were greater than 95.6%, and specificities were all greater than 99.8%.

The TV test on the GeneXpert (Xpert® TV assay) became available on the global market in 2017. Gaydos et al. reported on a multi-centre study involving 1867 eligible patients. The study prospectively collected urine, endocervical swabs and patient-collected vaginal swabs from female subjects presenting with symptoms associated with TV infection (714) and subjects who were asymptomatic (1153) [20]. The location of where the GeneXpert testing occurred was not specified. The authors reported the sensitivity for TV testing on the GeneXpert was 96.4% for self-collected vaginal swabs, 98.9% for endocervical specimens and 98.4% for female urine; while for men, sensitivity with urine samples was 97.2%. The specificity for TV on the GeneXpert for all specimen types was greater than 99%.

In 2018, a preliminary laboratory-based evaluation of the TV assay on the GeneXpert was conducted in Australia involving 120 urine samples, collected during routine remote community screenings in far north Queensland (60 positive and 60 negative) [21]. The authors reported the sensitivity of the TV assay on the GeneXpert was 95% and the specificity 100%.

The same year, the World Health Organization (WHO) also conducted a laboratory evaluation of the CT, NG and TV assays on the GeneXpert, using 339 samples spiked with phenotypically and genetically diverse strains of CT, NG and TV and other related species that may cross-react. Similar to other studies, the WHO study found high analytical sensitivity and specificity not only for CT and NG, but also for TV testing in the laboratory setting. No cross-reactivity was detected in the Xpert CT/NG or TV tests when testing samples of other causes of vaginal discharge such as bacterial vaginosis and Candida species. High rates of false positives for TV were identified when challenged with high concentrations of *Trichomonas tenax, Trichomonas gallinae, Trichomonas stableri*, and *Trichomonas aotus*); however, these species are not found in urogenital samples from humans, but rather inhabit the oral cavity of other animal species such as dogs, cats and birds and monkeys [22].

Following on from these limited studies, this present multi-country validation study of the GeneXpert for CT, NG and TV POC testing in primary care settings, led by the WHO, provides the first inter-country assessment of this molecular-based technology.

While the total study cohort numbered in excess of 1380 women considered at risk of STIs, there were large differences between countries in the percentage of patients recruited (with more than half from South Africa); the median age profile of patients (ranging from 22 years in South Africa to 40 in Morocco); and infection prevalence (for CT, from 20% in Morocco and Guatemala (Site 1) to 38% in South Africa; for NG, from 2% in Morocco and Guatemala (Site 1) to 20% in South Africa, and, for TV, from 15% in Guatemala (Site 1) to nearly 40% in Morocco). This wide variability in key study parameters (most notably the combination of both low and high prevalence sites) enhances, to some extent, the generalisability of study findings.

With regard to the acceptability of POCT, around 70% of participants were comfortable waiting at the clinic for their POC test results post analysis on the GeneXpert; however, a limitation of this study was intention to wait was not then directly linked to time to treat from receipt of that POC test result. Further, only 25% of participants were prepared to wait for between 1 and 2 h (the current turnaround time for either a TV or CT/NG result on the GeneXpert). At best, a POC test of even shorter turnaround time (for example 30 min to result) or an early termination assay for a detected pathogen would enable patients to access their results sooner and shorten the overall waiting time even further. Here lies a further challenge for device manufacturers.

Differences in the training system employed between countries may have contributed to the variability in performance (particularly sensitivity). As can be seen from Table 1, the health professional status of the POCT operator was variable; for example, a lab technician performed the test in Guatemala, a sexual health co-ordinator (highly qualified regional nurse) conducted the testing in Australia, and nurses undertook the testing in South Africa. In terms of delivery of training between countries, training in Australia was conducted by a senior scientist from the supporting institution – the Flinders University International Centre for Point-of-Care Testing, which was responsible for the design of the training package for the multi-country study; whereas in Guatemala and South Africa training was delivered by a local scientific representative from Cepheid (either in-person [Site 1] or on-line [Site 2]). Translation of the training resources to native language other than English was required in some countries (e.g. Guatemala). Despite the variable performance observed for patient test comparisons, concordance rates were excellent for the performance of quality testing (quality control and quality assurance samples) conducted in Australia, Morocco, Guatemalsa (Site 1) and South Africa before and across the COVID pandemic period as part of the on-going analytical surveillance maintained throughout the duration of this study; QC concordance was greater than 97% for all three tests and EQA concordance greater than 94% for each test reported by the countries involved; however a high rate of unsuccessful tests was reported for TV, most likely due to lack of stability with these quality products [14].

Interestingly, a significant difference in sensitivity for TV was observed between Guatemala Site 2 and Guatemala Site 1. As mentioned, these two data sets from the same country were separately analysed as there was no quality surveillance undertaken or available during the post-pandemic period when Guatemala Site 2 began recruiting participants and conducting POC testing. Without standardised quality surveillance which operated in the main study, it is not possible to know with any confidence whether tests performed were ‘in control’ or whether lot numbers of test cartridges were stable or stored and transported correctly. This highlights the critical need to include QC and EQA testing as part of a working POCT program because, without this element, there can be no confidence in the diagnostic accuracy of patient results produced.

It is unlikely that the logistics of patient sample transport to the reference laboratory resulted in degradation/instability of these samples for analysis. For example, samples were transported over 2,700 km and 1,400 km to the reference laboratories in Australia and South Africa, respectively. If this was a factor, one would expect that a sample reported as positive by POCT may have been reported as negative by the reference laboratory. However, the opposite was found, whereby samples that were negative by POCT were reported as positive by the laboratory (that is, the POCT test recorded higher levels of false negative results, hence poor sensitivity).

Differences in the limits of detection for the GeneXpert POCT method and the reference NAATs may be a potential factor in accounting for the discrepant results. A sub-study of the South African dataset investigated positive samples for CT, NG and TV with GeneXpert cycle threshold (Ct) values] greater than 35 [23]. For CT, 8% of the 275 samples that were true positives had Ct values > 35, while 13% of 15 false positive samples had Ct values more than 35. For NG, 2% of 141 true positive samples had Ct values for both gene targets greater than 35, while 30% of 10 false positive samples had Cts above 35. For TV, 4% of 82 true positives had Cts greater than 35 and 67% of 6 false positives had Cts higher than 35.

The presence of blood either in the samples tested or at the site of collection may be a potential interferent with the GeneXpert assay and was similarly investigated in a South African sub-study by the WHO team (Table 5). A sensitivity analysis of blood-stained samples (*n* = 133) indicated that, for all three tests, sensitivity estimates were better in blood-stained samples compared to those without the presence of blood (possibly as there were more symptomatic cases in this subgroup); in contrast, specificity estimates in the blood-stained samples were worse than those samples with no blood present (possibly due to interference by blood).

**Table 5:** Effect of blood on sensitivity and specificity estimates (plus 95% confidence intervals) in a subset of South African samples

Test	Measure	Blood-stained sample	
		Yes	No
Chlamydia	Sensitivity	94.4% (84.6–98.9)	85.63 (81.5–89.2)
	Specificity	96.6% (89.6–99.2)	97.56 (96.2–98.5)
Gonorrhoea	Sensitivity	100% (84.6–100)	89.04 (82.8–93.6)
	Specificity	96.4% (91.1–99.0)	99.18 (98.4–99.6)
Trichomoniasis	Sensitivity	71.4% (51.3–86.8)	63.57 (57.5–69.3)
	Specificity	96.5% (91.3–99.0)	99.22 (98.4–99.7)

In summary, despite the limitation of small sample numbers in some sites (Australia and Guatemala Site 1), this study provides the first major multi-country assessment of the analytical and clinical performance of primary care based GeneXpert POCT versus the reference laboratory NAATs. The sensitivity estimates observed, particularly for CT in Morocco (70.1%), and TV in Morocco (59.7%) and South Africa (66.1%), were lower than expected and lower than those previously published. Specificity estimates, on the other hand, were excellent (above 96%) for all three tests and across all countries involved. Pooled positive likelihood ratios for CT, NG and TV were all substantially greater than 10, also providing strong evidence that the GeneXpert POC test results were clinically adequate for ruling in the presence of infection. The pooled negative likelihood ratio for NG (0.10) was at, but not less than 0.10, the value referenced for the test to sufficiently rule out infection; however, the pooled negative likelihood ratios for CT and TV infection were much higher than 0.10, particularly for TV. In this scenario, the use of a second laboratory test to identify false negative POC test results is not viable nor affordable in either low- or high-income settings. It is important to note that the main objective of the ProSPeRo study was to evaluate point-of-care diagnostic technologies and conclude on their potential clinical usefulness under certain real-world scenarios.

## Conclusion

This multi-country evaluation of STI POCT is the first of its kind undertaken globally. Positive likelihood ratios and specificity estimates indicate the GeneXpert POC test results for CT, NG and TV were clinically acceptable for ruling in the presence of disease. However, negative likelihood ratios and variable sensitivity estimates from this study were poorer than expected for ruling out these infections, particularly TV.

## Data Availability

The data set analysed during the current study is available from the ProSPeRo study co-ordinators (KB and IT) upon reasonable request.

## Abbreviations

STI: Sexually transmitted infections
CT or Chlamydia: *Chlamydia trachomatis*
NG or Gonorrhoea: *Neisseria gonorrhoeae*
TV or Trichomoniasis: *Trichomonas vaginalis*
POCT: Point-of-care testing
POC test: Point-of-care test
NAAT: Nucleic acid amplification tests
LMIC: Low- and middle-income countries
AMR: Antimicrobial resistance
LR+: Positive likelihood ratio
LR-: Negative likelihood ratio
